# Sex differences in skeletal muscle Phosphatase and tensin homolog deleted on chromosome 10 (PTEN) levels: A cross-sectional study

**DOI:** 10.1038/srep09154

**Published:** 2015-03-17

**Authors:** M. Constantine Samaan, Sonia S. Anand, Arya M. Sharma, Imtiaz A. Samjoo, Mark A. Tarnopolsky

**Affiliations:** 1 Department of Pediatrics, McMaster University, Hamilton, Ontario, Canada; 2 Division of Pediatric Endocrinology, McMaster Children's Hospital, Hamilton, Ontario, Canada; 3 Population Genomics Program, Chanchlani Research Centre, McMaster University, Hamilton, ON, Canada; 4 Population Health Research Institute, Hamilton Health Sciences and McMaster University, Hamilton, Ontario, Canada; 5 Department of Medicine, McMaster University, Hamilton, Ontario, Canada; 6 Department of Clinical Epidemiology/Biostatistics, McMaster University, Hamilton, Ontario, Canada; 7 University of Alberta, Edmonton, Alberta, Canada

## Abstract

Women have higher adiposity but maintain insulin sensitivity when compared to men. Phosphatase and tensin homolog deleted on chromosome 10 (PTEN) inhibits insulin signaling, but it is not known if PTEN regulate insulin resistance in a sex-specific manner. In this cross-sectional study, muscle biopsies from participants in the Molecular Study of Health and Risk in Ethnic Groups (Mol-SHARE) were used to test for sex differences in PTEN expression. Quantitative real-time PCR was performed to determine PTEN gene expression (n = 53), and western blotting detected total and phosphorylated PTEN protein (n = 36). Study participants were comparable in age and body mass index. Women had higher fat mass percentage compared to men (40.25 ± 9.9% in women versus 27.6 ± 8.8% in men; mean difference −0.18, 95%CI (−0.24, −0.11), p-value <0.0001), with similar HOMA-IR (2.46 ± 2.05 in men versus 2.34 ± 3.06 in women; mean difference 0.04; 95% CI (−0.12, 0.21), p-value 0.59). Women had significant downregulation of PTEN gene expression (p-value 0.01) and upregulation of PTEN protein phosphorylation (inactivation) (p-value 0.001) when compared to men after correction for age, ethnicity, HOMA-IR, fat mass and sex. We conclude that the downregulation of muscle PTEN may explain the retention of insulin sensitivity with higher adiposity in women compared to men.

One of the main hallmarks of obesity is the deposition of excess fat in depots within and outside the adipose tissue^1^, and the increased adiposity seen in obesity is associated with skeletal muscle insulin resistance^2^. As skeletal muscle is the main organ responsible for postprandial glucose disposal^3^, its insulin resistance is a major contributor to the global epidemic of type 2 diabetes^4^,^5^,^6^,^7^. Understanding the mechanisms driving muscle insulin resistance is key to treating and preventing type 2 diabetes, as it will allow the implementation of specific interventions designed to restore insulin sensitivity.

One of the factors that determine adiposity patterns is sex. It is well known that women have a higher fat mass when compared to men^8^. Importantly, despite women having higher adiposity, this does not appear to adversely impact insulin sensitivity when compared to men at a given weight^9^,^10^. This important observation may be explained by sex-based differential expression of molecules that regulate the insulin-signaling pathway.

One of the molecules that regulate muscle insulin signaling is Phosphatase and tensin homolog deleted on chromosome 10 (PTEN)^11^,^12^. PTEN inhibits insulin-stimulated Phosphatidylinositol-3-kinase/Akt (PI3K/Akt) signaling, and is reported to be upregulated in muscle of obese mice^13^. It is not known whether sex differences in muscle PTEN expression contribute to equivalent insulin sensitivity despite higher adiposity in women when compared to men.

We hypothesized that lower muscle PTEN expression levels results in the relative retention of insulin sensitivity despite higher adiposity in women compared to men.

## Results

The study group included 34 women and 42 men, and gene expression data were available on 53 (n = 21 female) and protein data on 36 participants (n = 15 female). Men and women were of similar age and had similar body mass index (BMI) (Table 1).

Of note, four women were on oral contraceptive therapy, and one was on estrogen therapy.

### Women have higher fat mass compared to men

When comparing body composition between women and men, women had significantly higher fat mass percentage, which was mainly due to higher superficial subcutaneous fat in comparison to men. On the other hand, men had higher lean mass and waist-to hip ratio when compared to women (Table 2). There were no differences between men and women in HOMA-IR (2.46 ± 2.05 in men versus 2.34 ± 3.06 in women, mean difference 0.04; 95% CI (−0.12, 0.21)) (Table 3).

### Muscle PTEN gene expression is lower in women compared to men

**Figure 1:**
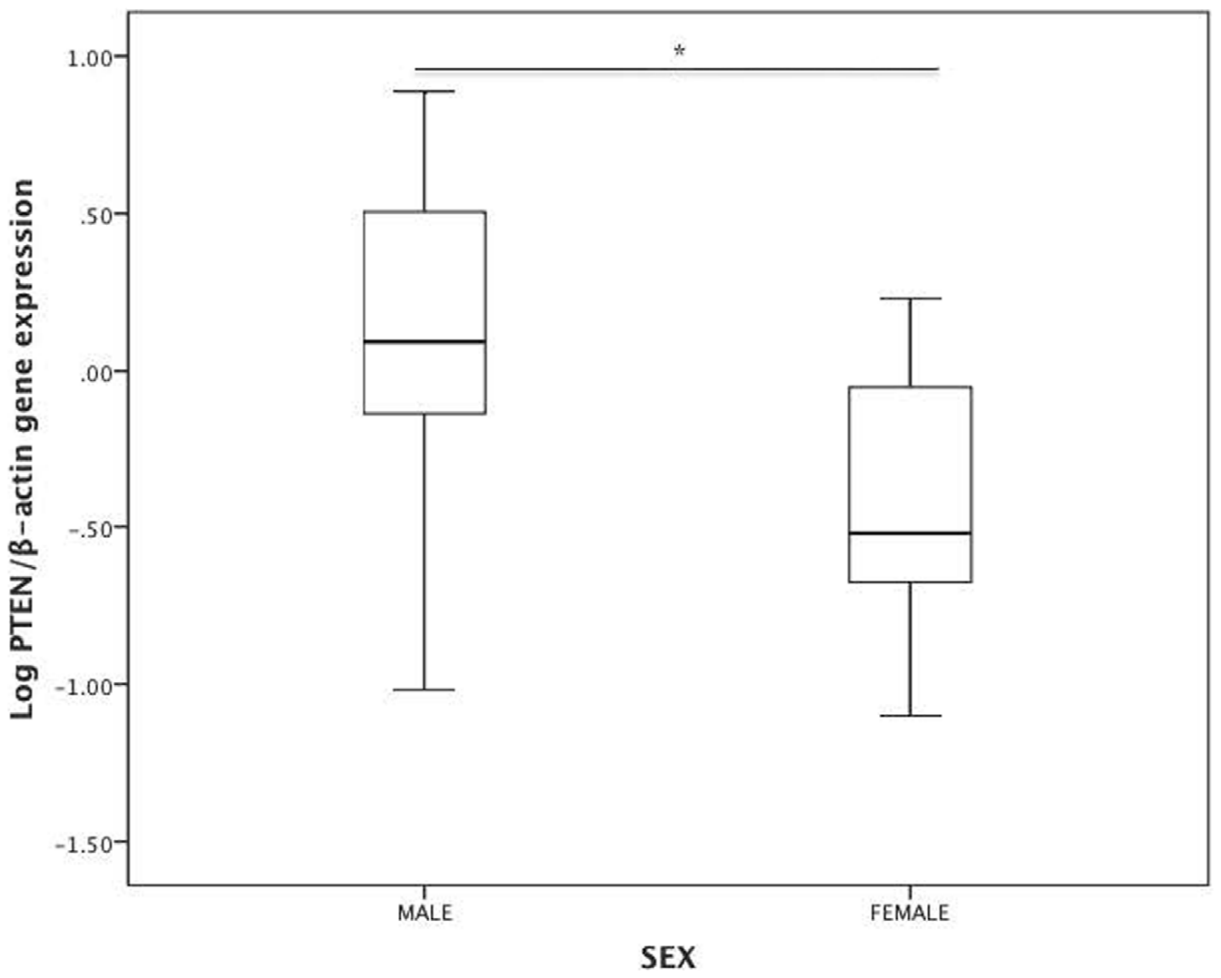
Log PTEN/β-actin (n = 53) gene expression in men and women.

To test whether there are sex differences in muscle PTEN levels, we first analyzed PTEN gene expression levels in muscle using Quantitative Real-Time PCR (qRT-PCR). In the unadjusted analysis, PTEN gene expression was significantly lower in women when compared to men (Figure 1, p-value < 0.0001).

This lower muscle PTEN gene expression in women persisted after adjustment for age, ethnicity, fat mass percentage, and log HOMA-IR (Table 4, β −0.31; 95% CI (−0.54, −0.08), p-value 0.01).

### Total muscle PTEN protein expression is similar in women & men

In order to determine if the reduction in gene expression in women is associated with reduced PTEN protein levels, we performed western blot analysis on muscle lysates from men and women.

**Figure 2:**
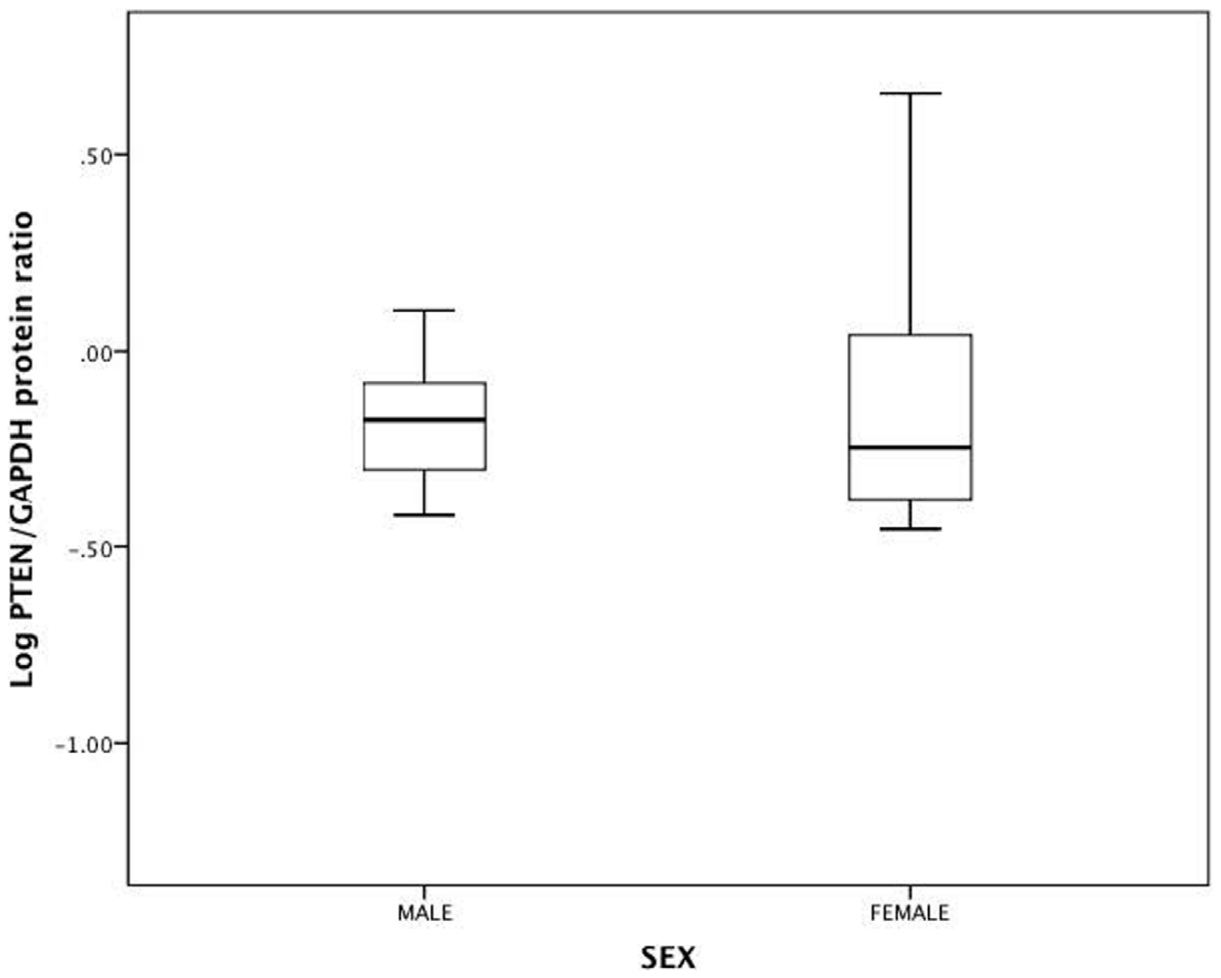
Log PTEN/GAPDH protein ratio (n = 36) in men and women. GAPDH = glyceraldehyde 3-phosphate dehydrogenase.

PTEN gene expression did not correlate with PTEN protein levels in muscle (p-value 0.35). However, unadjusted normalized total PTEN protein levels (PTEN/GAPDH) were similar in men and women (Figure 2, p-value 0.2), and this remained after adjustment for age, ethnicity, fat mass percentage, and log HOMA-IR (Table 4, β 0.39; 95% CI (−0.08, 0.87), p-value 0.1).

### Women have higher muscle PTEN protein phosphorylation (inactivation) compared to men

**Figure 3:**
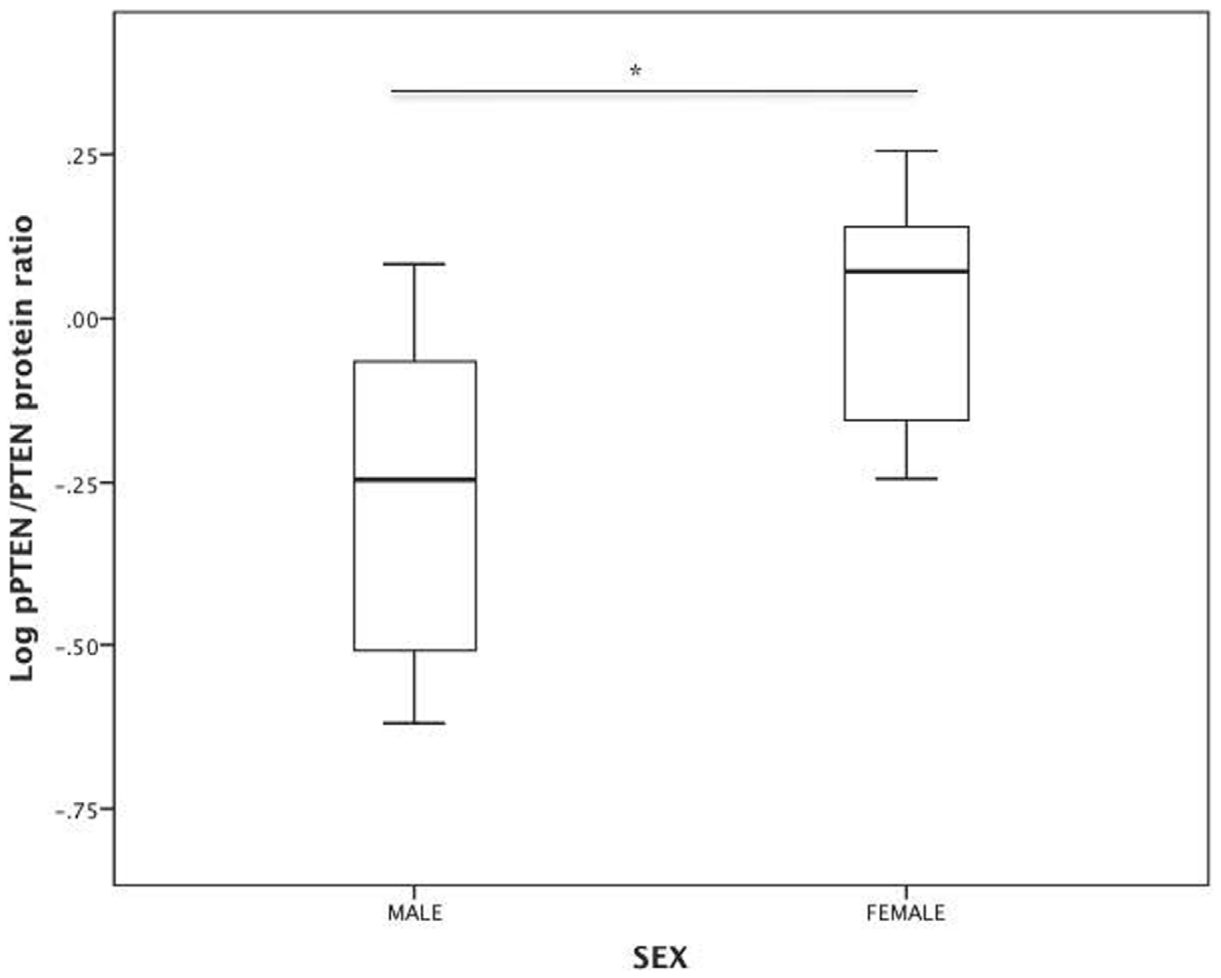
Log pPTEN/PTEN protein ratio (n = 36) in men and women.

**Figure 4:**
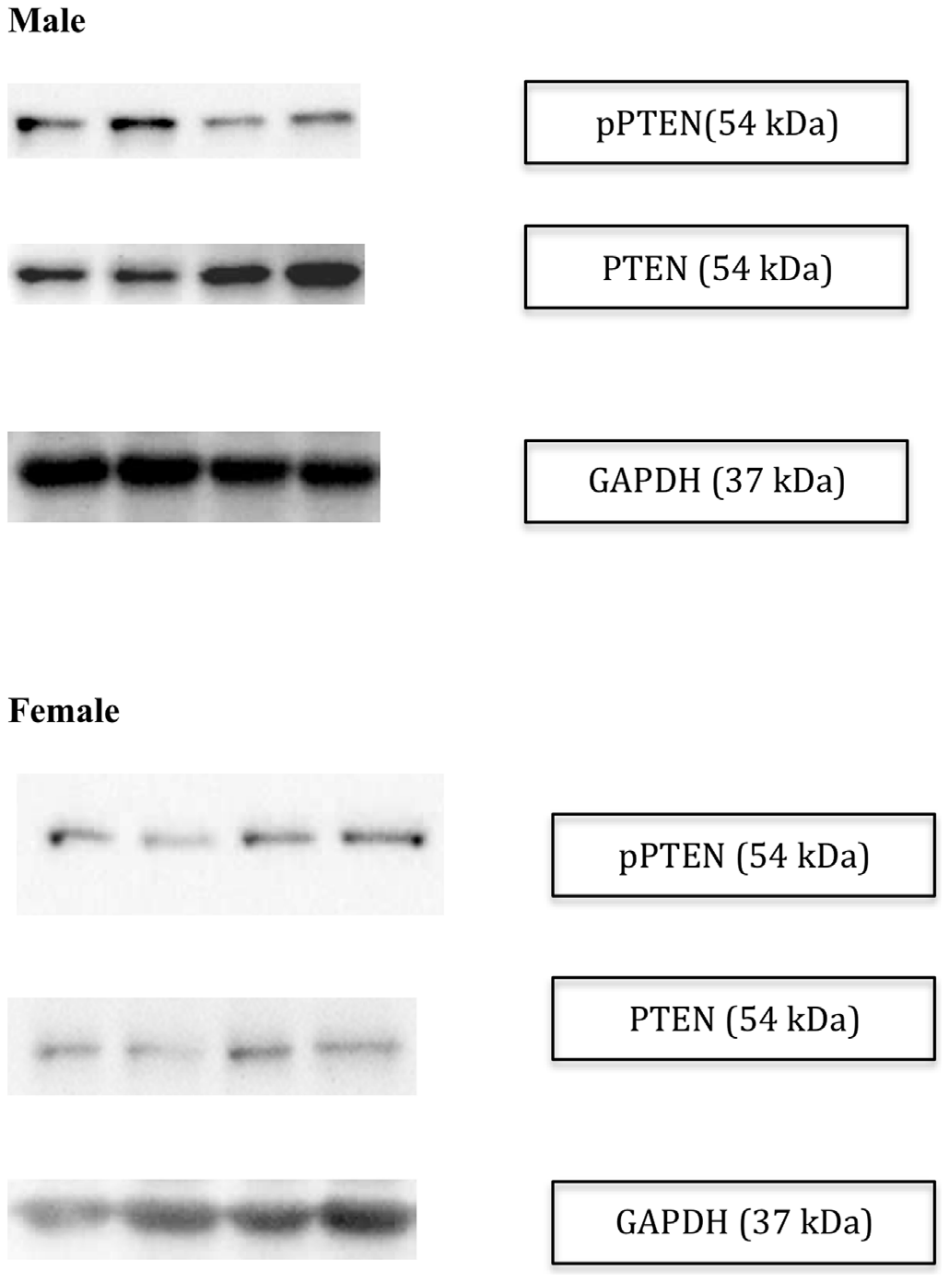
Representative images for western blot data from (a) male and (b) female participants.

To determine if there were differences in PTEN protein activity, we measured the phosphorylated (inactivated) version of PTEN protein. Women had higher pPTEN/PTEN ratio (Figure 3, unadjusted analysis p-value 0.002; Figure 4), and this persisted with adjustment for age, ethnicity, fat mass percentage, and log HOMA-IR (Table 4, β 0.85; 95% CI (0.38, 1.32), p-value 0.001). This higher pPTEN/PTEN ratio indicates the presence of more inactive PTEN protein in muscle of women when compared to men.

## Discussion

At similar BMI levels, women maintain their insulin sensitivity when compared to men despite having higher adiposity^9^. In this study, we investigated the sex differences in muscle PTEN gene expression, protein content and activity to see if PTEN downregulation is involved in this paradox.

We demonstrate that women have lower muscle PTEN gene expression when compared to men, despite having higher adipose tissue mass. This is coupled with increased inactivation of PTEN protein.

PTEN is a dual protein and lipid phosphatase that interferes with the insulin-signaling pathway via its lipid phosphatase activity. PTEN itself can be inactivated by phosphorylation^14^,^15^,^16^, and this post-translational modification impact PTEN activity. PTEN can autoinhibit itself through S380-385 sites, whereby phosphorylation of S385 leads to the phosphorylation of S380 and Threonine sites, and binding of the COOH tail to the C2 and phosphatase domains, preventing the binding of PTEN to a complex of protein that drive its activity^17^.

In addition, Casein Kinase II (CK2) phosphorylates PTEN (S370, S385), but the biological importance of this is uncertain^18^. CK2 also seem to prime certain sites (S362, T366) for phosphorylation via the glycogen synthase kinase-3β (GSK3β) pathway. The latter sites form a feedback loop to inhibit growth factor signaling via the PI3K pathway and PTEN. Interestingly, neither CK2 nor GSK3β affect S380 phosphorylation^19^.

In addition, RhoA-associated kinase (ROCK), acting on the C2 domain of PTEN, upregulate leukocyte chemotaxis via phosphorylation and activation of S229, T232, T319, and T321 sites^20^. In contrast, PI3K p110δ subunit inactivates PTEN in macrophages through inhibition of RhoA/ROCK^21^.

In addition, it has been shown that leptin plays an important role in phosphorylation (inhibition) of PTEN in the hypothalamus^22^, but the significance of this is uncertain.

The PTEN-mediated downregulation of insulin signaling may be explained by the presence of negative feedback loops in the insulin signaling pathway itself that become activated with increased adiposity, including Forkhead box O (FOXO) proteins and Mammalian Target Of Rapamycin Complex 1 (mTORC1) and its downstream effector S6K 1 and 2^23^,^24^,^25^,^26^.

In obese mice with normal ability to express PTEN, there is upregulation of muscle PTEN protein that is associated with reduced insulin signaling^13^. In addition, muscle-specific PTEN knockout mice have enhanced insulin sensitivity when rendered obese^27^. In contrast, transgenic overexpression of PTEN in mice leads to hyperphagia yet lower adiposity. Interestingly, this is coupled with reduced insulin signaling but maintained insulin sensitivity. This maintenance of insulin sensitivity in transgenic mice is due to increased brown adipose tissue activity, which promotes energy expenditure and lowers nutrient storage^28^,^29^.

In humans, polymorphisms of *PTEN* gene leading to higher PTEN expression levels have been noted in diabetes^30^. In contrast, *PTEN* haploinsufficiency seen in Cowden syndrome, a cancer predisposition syndrome, is associated with obesity and paradoxical enhancement of insulin sensitivity^31^. Taken together, the above lines of evidence suggest that PTEN has direct and indirect effects on insulin sensitivity and signaling in different organs in rodents and humans.

In our study, the downregulation of PTEN gene expression and PTEN protein inactivation in women may protect against the inhibition of PI3K/Akt signaling with increased adiposity. As low levels of Akt activity are needed to maintain maximal glucose uptake^25^, even relatively small reductions in PTEN activity can result in maintained insulin action despite higher adiposity levels in women when compared to men.

The sex differences in muscle insulin sensitivity may be explained by differences in sex steroids^32^,^33^. Estrogen, mainly a female hormone, stimulates muscle Akt signaling and glucose transporter 4 gene expression independently of insulin^32^,^33^. In addition, post-menopausal women have reduced insulin sensitivity that improves with estradiol therapy^34^, and insulin resistance was noted in men with defects in estrogen synthesis or response^35^,^36^. The mechanisms through which estrogen may interact with PTEN need to be clarified in future studies.

The strengths of this study include the relatively large sample size of muscle biopsies from well-characterized study participants, and the detailed characterization of PTEN at gene and protein expression levels.

We did not study the effects of insulin stimulation on PI3K/Akt pathway to correlate this with PTEN expression, as we did not treat the muscle tissue with insulin prior to freezing, which is a limitation of this study. In addition, we did not study adipose tissue insulin signaling or glucose uptake. In mice with adipose-specific PTEN deletion, increased adipose tissue insulin sensitivity was associated with reduced muscle insulin sensitivity, which may be an attempt to maintain whole body insulin sensitivity^37^.

In summary, this study shows that muscle PTEN is regulated in a sex-specific manner, and makes PTEN an attractive therapeutic target in treatment and prevention of insulin resistance and type 2 diabetes.

## Methods

The samples used in this report were from the Molecular Study of Health and Risk in Ethnic Groups *(Mol-SHARE study).* This study was designed to understand the mechanisms underlying ethnic variations in predisposition to adverse cardiometabolic outcomes, and compared South Asians to Europeans (ClinicalTrials.gov Identifier NCT00249314). The study recruited participants between 18–50 years of age, and study procedures and measurements have been reported previously^38^. Total fat mass (FM) was measured using DXA scans after an overnight fast. Subcutaneous (SAT) and visceral adipose tissue (VAT) compartments were measured using MRI of the abdomen by attaining T1-weighted MRI image at the level of mid-L4 (TR 400 ms, TE 13 ms). The volume of SAT and VAT was determined by manual tracing of the specific fat depot.

The Hamilton Integrated Research Ethics Board approved the study, and all participants provided written informed consent. This study is utilizing a subset from the full study that has muscle biopsy samples available. The study was conducted in accordance with current clinical practice guidelines and legislation.

We used BMI cutoff points including 18.5–24.9 kg/m^2^ for normal weight, 25–29.9 kg/m^2^ for overweight, and ≥30 kg/m^2^ to classify participants. In this analysis, we grouped subjects to maximize statistical power, and log-transformed values of HOMA-IR was used to provide a measure of insulin resistance.

### Metabolic biomarkers

Study participants provided blood samples after an overnight fast (12 hours). Fasting serum lipid profile was generated using enzymatic methods for cholesterol^39^, while serum LDL was calculated using the Friedewald formula^40^, and HDL was quantified with a homogenous enzymatic colorimetric assay (ROCHE/Hitachi Modular Package Insert). Glucose was measured using the hexokinase/glucose-6-phosphate dehydrogenase method^41^. Triglycerides were quantified with an enzymatic colorimetric assay (ROCHE/Hitachi Modular instrument and reagent kit). Insulin was quantified by an electrochemiluminescence immunoassay using the Roche Elecsys R 2010 immunoassay analyzer (Roche Diagnostics GmbH, Indianapolis, Indiana, USA). Insulin resistance was determined by calculating the homeostatic model assessment-insulin resistance (HOMA-IR)^42^,^43^.

### Muscle biopsy

Muscle biopsies were obtained from the vastus lateralis muscle under local anesthesia by a modified Bergstrom needle with suction^38^.

### Total RNA extraction

Trizol Reagent was purchased from Life Technologies and used in total RNA isolation. Muscle samples were homogenized in 1 ml Trizol with a power homogenizer on ice twice for 15 second interval at each attempt. The samples were mixed by inverting 4–5 times and placed at room temperature for 5 minutes. Then, 200 uL chloroform was added, and samples were shaken vigorously for 15 seconds and left at room temperature for 2–3 minutes. The samples were then spun down at 12,000 g, 4°C, for 15 minutes. The aqueous phase was transferred to new tubes and 500 uL isopropanol was added to the aqueous phase followed by a brief vortex. The samples were then left overnight at −20°C, and then centrifuged at 12,000 g, 4°C, for 15 minutes. The supernatant was decanted and 1 ml 70% ethanol added to the pellet and mixed with sample. Samples were then centrifuged at 7,500 g, 4°C, for 5 minutes. After air-drying the pellet, nuclease-free water was added to each tube and samples incubated at 55°C for 10 minutes. Samples were cooled down for 15 minutes at room temperature and RNA purity was measured with a spectrophotometer. RNA samples with 260/280 ratios at 1.8–2 were then used to synthesize cDNA.

### Reverse transcription reaction to generate cDNA

Reverse transcription reaction was performed using SuperScript® III First-Strand Synthesis kit (Invitrogen, Carlsbad, CA) following DNase treatment for 30 minutes at 37°C, and the reverse transcription reaction was conducted using 1 ug of RNA as template according to the manufacturer's instructions.

### Quantitative Real-Time PCR (qRT-PCR)

PTEN gene expression analysis was conducted in triplicates using the Rotor-Gene 6000 qRT-PCR machine (Corbett Research; Mortlake, Australia). We used TaqMan® Gene Expression Assays (Applied Biosystems; Foster City, CA) of either PTEN (TaqMan assay Hs02621230_s1) or beta-Actin as the endogenous control gene (TaqMan assay Hs01060665_g1). Statistical analysis of qRT-PCR data was performed using the ΔΔCt method^44^.

### Western blot

Quantification of PTEN and pPTEN muscle protein content was done using western blotting. Biopsies from vastus lateralis muscle were homogenized as previously described^45^, and 50 ug was loaded on an 8% polyacrylamide gel. Membranes were blocked in 5% BSA in TBST (0.1% Tween-20), and blots were incubated with PTEN, pPTEN^Ser380^ and GAPDH primary antibodies (Cell Signaling, dilution 1:1000) in 5% BSA in TBST. Anti-Rabbit IgG HRP-Linked (1:3000 dilution) in 5% BSA in TBST was used as secondary antibody. Amersham™ ECL™ Western Blotting detection reagent (GE HealthCare) was used to detect the protein signal, and ImageJ software was used to quantify the protein density with normalization of PTEN to GAPDH and pPTEN ^Ser380^ to total PTEN^46^.

### Statistical analysis

Data were tested for normality using Shapiro-Wilk test and log transformed if not normally distributed, and variance inflation factor was implemented to rule out colinearity. Independent sample t-test was used to compare the variables without adjustment, and two-tailed statistical significance results are reported. A general Linear Model was used in the analyses with PTEN gene expression, PTEN/GAPDH and PTEN/pPTEN as dependent variables and adjusting for age, fat mass percentage, HOMA-IR, ethnicity and sex as covariates. We report the mean differences between men and women and the respective 95% confidence intervals. Data are reported as mean ± SD unless otherwise stated, and significance was set at p-value of less than 0.05. SPSS version 22 was used for data analysis (Armonk, NY: IBM Corp).

## Author Contributions

M.C.S., M.A.T. and S.S.A. conceived the study hypothesis. M.C.S., M.A.T., S.S.A., A.M.S. and I.A.S. conceived and designed the experiments. M.C.S., M.K. and I.A.S. performed experimental work. M.C.S., M.A.T., S.S.A. and I.A.S. analyzed the data and M.C.S. wrote the first draft, and all authors provided feedback to the submitted version of the manuscript.

## Figures and Tables

**Table 1:** Clinical characteristics of the participants

	Male (n = 42)		Female (34)			95% Confidence Interval		
Variable	Mean	SD	Mean	SD	Mean Difference	Lower	Upper	P-value
**Age (years)**	34.64	10.22	34.76	10.68	0.0007	−0.06	0.07	0.98
**Height (cm)**	175.40	6.99	164.01	6.52	0.03	0.02	0.04	<0.0001
**Weight (kg)**	84.44	12.96	73.06	16.64	0.06	0.03	0.10	0.001
**BMI (kg/m^2^)**	27.26	3.98	27.35	6.32	0.004	−0.03	0.04	0.81
**Waist circumference (cm)**	96.24	12.51	88.36	12.70	0.036	0.01	0.07	0.02
**Hip circumference (cm)**	105.40	9.48	109.08	12.06	−0.014	−0.03	0.01	0.18
**Heart rate (bpm)**	64.79	11.68	65.68	7.47	−0.003	−0.04	0.03	0.86
**Systolic BP (mmHg)**	113.10	9.26	108.88	9.69	0.017	−0.001	0.04	0.07
**Diastolic BP (mmHg)**	74.38	9.15	72.68	6.53	0.005	−0.02	0.03	0.65
**Fat mass (kg)**	22.60	10.06	29.20	13.30	−0.31	−0.56	−0.05	0.02

bpm = beats per minute; BP = blood pressure.

**Table 2:** Characteristics of the lean and fat mass compartments in men and women

	Male (n = 42)		Female (34)			95% Confidence Interval		
Variable	Mean	SD	Mean	SD	Mean Difference	Lower	Upper	P-value
**% Fat mass**	27.6	8.8	40.25	9.86	−0.18	−0.24	−0.11	<0.0001
**WHR**	0.91	0.08	0.81	0.07	0.05	0.03	0.07	<0.0001
**Lean mass (kg)**	57.6	7	40.5	4.3	0.15	0.13	0.17	<0.0001
**SAT (cm^2^)**	224.9	103	277.44	139.97	−0.1	−0.2	0.01	0.069
**Superficial SAT (cm^2^) (n = 40 male, n = 28 female)**	100.57	33.71	137.93	76.36	−0.12	−0.22	−0.02	0.006
**Deep SAT (cm^2^) (n = 42 male, n = 31 female)**	152.44	83.47	173.67	123.42	−0.02	−0.13	0.09	0.56
**VAT (cm^2^)**	126.51	75.32	105.77	65.58	0.07	−0.06	0.21	0.27

WHR = waist-to-hip ratio; SAT = subcutaneous adipose tissue; VAT = visceral adipose tissue.

**Table 3:** Metabolic phenotype and PTEN gene and protein expression (n = 76 unless otherwise stated)

	Male		Female			95% Confidence Interval		
Variable	Mean	SD	Mean	SD	Mean Difference	Lower	Upper	P-value
**FBG (mmol/l)**	5.03	0.52	4.87	0.66	0.02	−0.01	0.04	0.18
**Fasting insulin (μIU/ml)**	10.68	8.01	13.29	20.69	−0.003	−0.16	0.16	0.97
**Cholesterol (mmol/l)**	4.75	1.06	4.64	0.88	0.007	−0.04	0.05	0.73
**Triglycerides (mmol/l)**	1.51	1.09	1.12	0.69	0.01	−0.02	0.21	0.11
**HDL (mmol/l)**	1.23	0.33	1.44	0.33	−0.08	−0.13	−0.02	0.01
**LDL (mmol/l)**	2.89	0.94	2.66	0.67	0.02	−0.05	0.1	0.52
**HOMA-IR**	2.46	2.05	2.34	3.06	0.04	−0.12	0.21	0.59
**Log PTEN gene expression (n = 53, SE)**	0.13	0.07	−0.44	0.09	0.57	0.33	0.8	<0.0001
**Log PTEN/GAPDH protein ratio (n = 36, SE)**	−0.26	0.07	−0.12	0.09	−0.14	−0.37	0.08	0.20
**Log pPTEN/PTEN protein ratio (n = 36, SE)**	−0.27	0.05	−0.001	0.06	−0.27	−0.43	−0.11	0.002

SD = standard deviation; SE = standard error; FBG = fasting blood glucose; HDL = high-density lipoprotein; LDL = low-density lipoprotein; HOMA-IR = homeostatic model assessment-insulin resistance; GAPDH = glyceraldehyde 3-phosphate dehydrogenase.

**Table 4:** The general linear model analysis of PTEN gene expression and PTEN/GAPDH and pPTEN/PTEN protein content in muscle

Gene expression data (Log PTEN/actin)				
Parameter	β	95% Confidence Interval		P-value
**Age/years**	−0.005	−0.02	0.01	0.36
**Ethnicity**	−0.13	−0.37	0.18	0.3
**Log HOMA-IR**	−0.15	−0.65	0.36	0.56
**Log %FM**	−0.58	−1.65	0.5	0.29
**Sex**	−0.43	−0.75	−0.11	0.01

Protein data (Log PTEN/GAPDH)				
Parameter	β	95% Confidence Interval		P-value
**Age/years**	0.003	−0.12	0.02	0.69
**Ethnicity**	−0.25	−0.49	−0.01	0.046
**Log HOMA-IR**	0.04	−0.4	0.48	0.85
**Log %FM**	−0.08	−0.94	0.78	0.85
**Sex**	0.24	−0.05	0.52	0.1

Protein data (Log pPTEN/PTEN)				
Parameter	β	95% Confidence Interval		P-value
**Age/years**	0.01	−0.01	0.02	0.27
**Ethnicity**	−0.08	−0.26	0.11	0.4
**Log HOMA-IR**	−0.15	−0.47	0.18	0.37
**Log %FM**	−0.48	−1.21	0.16	0.14
**Sex**	0.39	0.17	0.6	0.001

%FM = fat mass percentage.
